# Increased lateral and medial femoral posterior radius ratios are risk factors for anterior cruciate ligament injury

**DOI:** 10.1186/s12891-022-05052-y

**Published:** 2022-02-05

**Authors:** Chunxu Fu, Xuguo Fan, Shigang Jiang, Junsen Wang, Tong Li, Kai Kang, Shijun Gao

**Affiliations:** Department of Orthopaedic Surgery, Third Hospital of Hebei Medical University, 139 Ziqiang Road, Shijiazhuang, 050051 Hebei People’s Republic of China

**Keywords:** Knee, Anterior cruciate ligament injury, Knee joint kinematics, Computed tomography

## Abstract

**Background:**

Many studies have shown that distal femoral sagittal morphological characteristics have a clear relationship with knee joint kinematics. The aim of this study was to determine the relationship between distal femoral sagittal morphological characteristics and noncontact anterior cruciate ligament (ACL) injury.

**Methods:**

A retrospective case-control study of 148 patients was conducted. Two age- and sex-matched cohorts (each *n* = 74) were analysed: a noncontact ACL injury group and a control group. Several characteristics were compared between the two groups, including the lateral femoral posterior radius (LFPR), medial femoral posterior radius (MFPR), lateral height of the distal femur (LH), medial height of the distal femur (MH), lateral femoral anteroposterior diameter (LFAP), medial femoral anteroposterior diameter (MFAP), lateral femoral posterior radius ratio (LFPRR), and medial femoral posterior radius ratio (MFPRR). Receiver operating characteristic (ROC) analysis was used to evaluate the significance of the LFPRR and MFPRR in predicting ACL injury.

**Results:**

Compared with patients in the control group, patients in the ACL injury group had an increased LFPR, MFPR, MFAP, LFPRR, and MFPRR. ROC analysis revealed that an increased LFPRR above 31.7% was associated with noncontact ACL injury, with a sensitivity of 78.4% and a specificity of 58.1%; additionally. an increased MFPRR above 33.4% was associated with noncontact ACL injury, with a sensitivity of 58.1% and a specificity of 70.3%.

**Conclusion:**

This study showed that increased LFPRR and increased MFPRR are risk factors for developing noncontact ACL injury. These data could thus help identify individuals susceptible to ACL injuries.

## Background

Injuries to the anterior cruciate ligament (ACL) are debilitating [1, 2] and are becoming increasingly common among active individuals [3–5]. Although the mechanism leading to ACL injury has not been established, identifying risk factors help prevent noncontact ACL injury and achieve optimal outcomes in ACL reconstruction. Many studies have investigated distal femoral osseous morphological characteristics as risk factors for noncontact ACL injury [6], including an A-shaped notch [7], decreased notch width index [8, 9], smaller femoral notch volume [10], and increased thickness of the medial intercondylar ridge [8]. These osseous morphological characteristics have been shown to have a significant association with noncontact ACL injury.

Conventional radiographs have been used to characterize distal femoral sagittal morphology to determine its relationship with ACL injury. Increased lateral posterior femoral condylar depth [11] and a decreased ratio of lateral femoral condylar height to anteroposterior diameter [12] are reported risk factors for noncontact ACL injury. However, these previous studies did not investigate the osseous morphological characteristics of the lateral and medial condyles separately, and they overlooked the difference between the lateral femoral condyle and medial condyle [13, 14], the presence of which has been confirmed. Additionally, the relationship between the distal femoral sagittal morphology and knee joint kinematics has been ignored. Several studies have reported that the femoral posterior condyles have a single radius in the arc that articulates with the tibia from 10° to 160° and that this single radius defines a single axis that represents the flexion-extension axis of the knee [15, 16]. Most flexion of the knee occurs on the femoral posterior condyles [17–19], the area at which most noncontact ACL injuries also occur. These sagittal osseous morphological characteristics of the distal femoral condyle that are associated with knee joint kinematics might affect the occurrence of noncontact ACL injury. Despite previous studies, it remains unclear whether the lateral and medial sagittal osseous morphological characteristics of the distal femur are associated with noncontact ACL injury.

The objective of this study was to determine whether lateral and medial distal femoral sagittal morphology, which is associated with knee joint kinematics, is associated with noncontact ACL injury. It was hypothesized that there are specific osseous morphological characteristics that are associated with noncontact ACL injury.

## Materials and methods

### Subjects

After hospital institutional review board approval was obtained, the medical records of patients treated in our hospital between 2019 and 2021 were retrospectively reviewed. Eligibility for this study required patients to have computed tomography (CT) data available for the injured knee. The patients were categorized into one of two groups: (1) those with a noncontact ACL injury and (2) those who had a fracture of the tibial plateau resulting from a violent injury (control group). To be included as a case in the ACL injury group, patients were confirmed via clinical examination, magnetic resonance imaging (MRI), and arthroscopic visualization at the time of ACL reconstruction by two experienced orthopaedic surgeons. A noncontact ACL injury was defined as an event not occurring due to direct contact between the ACL-injured knee and the ground, another athlete, or other object.

**Fig. 1:**
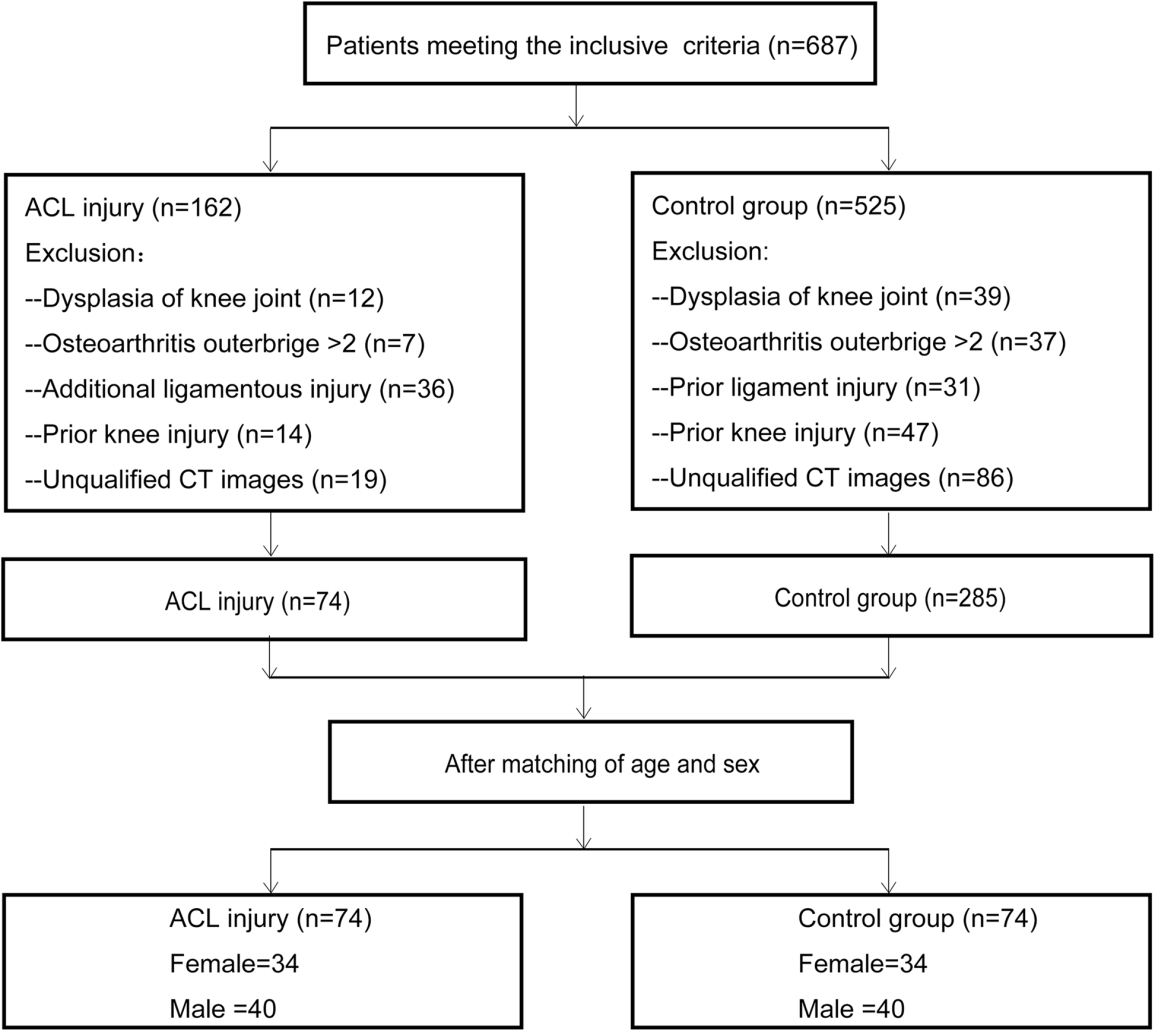
Flow diagram of patient enrolment

Our inclusion criteria were as follows: noncontact ACL injury or fracture of the tibial plateau, CT scan for the injured knee, 18–45 years old and having a body mass index (BMI) between 18 and 45 kg/m^2^. Our exclusion criteria were as follows: dysplasia of the knee joint, evidence of osteoarthritis, prior knee injury, or inadequate CT images (such as CT scans without intact femoral condyles). The patients were classified according to type of injury, either noncontact ACL injury or fracture of the tibial plateau. Subjects were excluded from the ACL injury group if they had additional ligamentous injury (medial collateral ligament, lateral collateral ligament, posterior cruciate ligament, or medial patellofemoral ligament). In addition, tibial plateau fractures were also excluded from the ACL group, such as ACL avulsion fractures, so that the ACL injuries were exclusively ACL body injuries. After the medical records were reviewed for eligibility, 74 noncontact ACL-injured cases (34 females, 40 males) were identified from the Department of Orthopaedics at our hospital. The control data were obtained from patients treated in the trauma centre of the same hospital and matched to ACL-injured patients by age and sex. Subjects were excluded from the control group if they had a prior ligament injury (medial collateral ligament, lateral collateral ligament, posterior cruciate ligament, and medial patellofemoral ligament). Although tibial plateau fractures are often associated with avulsion of the ACL, PCL, MCL and PLC, the mechanisms of noncontact ACL injuries are different from those of tibial plateau fractures, which are high-energy violent injuries. The control group was composed of 74 individuals (34 females, 40 males). Figure 1 shows the flow diagram of patient enrolment in the study.

### Three dimensional model reconstruction

CT scanning was performed using a 64-slice CT scanner (Somatom Sensation 64, Siemens, Erlangen, Germany) with the knee in extension following surgery to evaluate surgical outcomes. To obtain an accurate sagittal view, a three-dimensional model of the distal femur was created with Digital Imaging and Communications in Medicine (DICOM) CT images, which were obtained using the image processing software Mimics (21.0 Materialise, Leuven, Belgium). The threshold of all cases was set at 226 HU, and the femoral mask was automatically separated using the “Region Grow” function. The three-dimensional model of the femur was reconstructed using the “Calculate Part” function, and the optimal quality was chosen. Then, three-dimensional rotation of the femoral model was performed using the “Pan” and “Rotate” functions for accurate realignment. To obtain the nonorthogonal, sagittal imaging plane, rotation of the femoral three-dimensional model was performed as described by Howell et al. [16]. This was defined as the sagittal imaging plane of the distal femur. The sagittal imaging plane of the medial distal femur was considered to be plane a, and the sagittal imaging plane of the lateral distal femur was considered to be plane b.

### Measurement methods

**Fig. 2:**
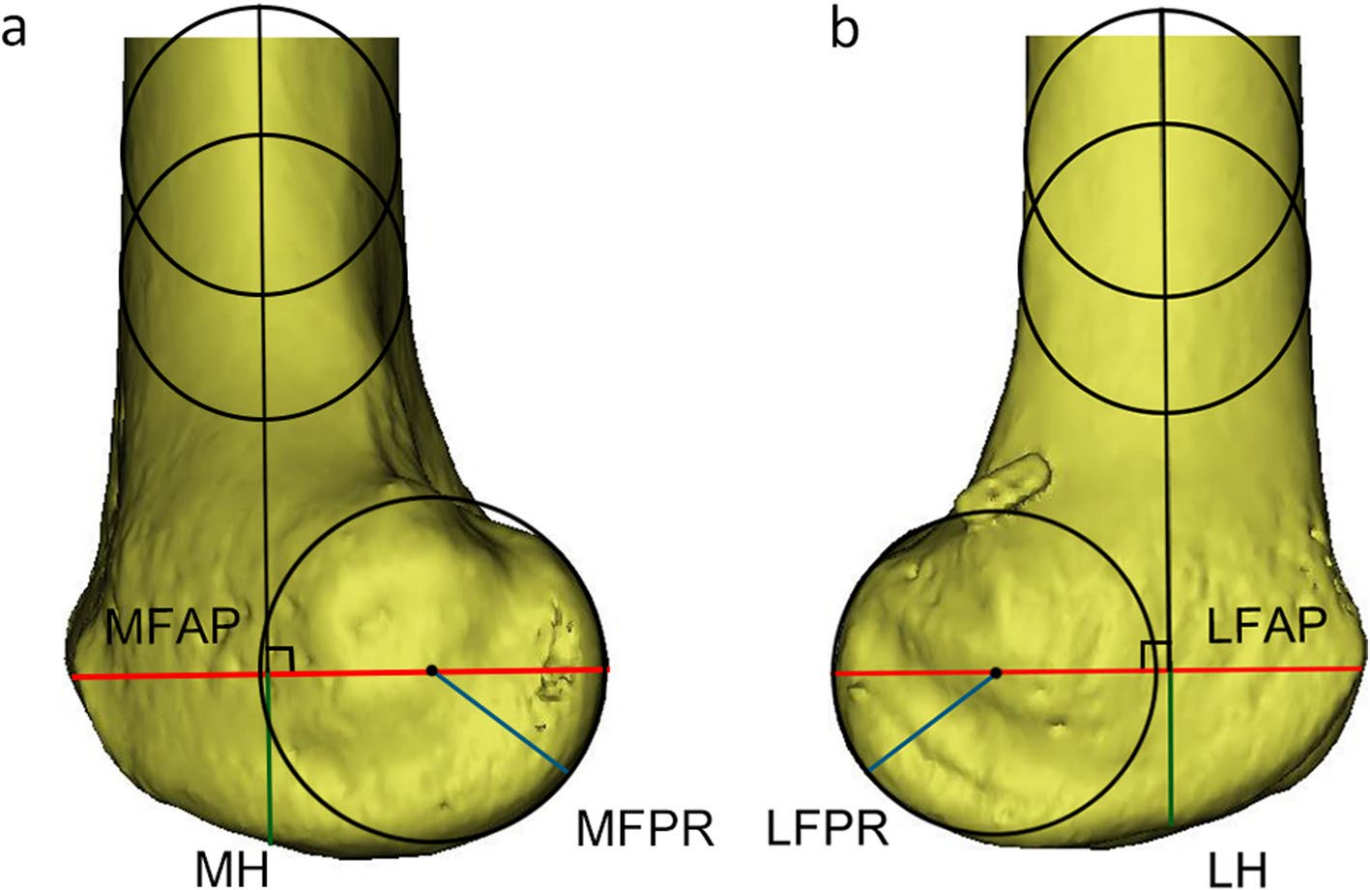
The sagittal imaging plane showing the measurements. **a** Sagittal imaging plane of the medial femoral condyle. **b** Sagittal imaging plane of the lateral femoral condyle. The long axis of the femoral shaft is determined by a line through the centres of two circles centred on the femoral shaft. The best fit circle to the posterior curvature of the femoral condyle determines the lateral femoral posterior radius (LFPR) and medial femoral posterior radius (MFPR) (blue line). A line passing through the centre of the posterior circle and perpendicular to the long axis of the femoral shaft determines the lateral femoral anteroposterior diameter (LFAP) and medial femoral anteroposterior diameter (MFAP) (red line). The distance from the intersection of these lines to the distal femur determines the lateral height of the distal femur (LH) and the medial height of the distal femur (MH) (green line)

Measurements for both study groups were obtained from a sagittal view image by two independent blind observers and consisted of the lateral femoral posterior radius (LFPR), medial femoral posterior radius (MFPR), lateral height of the distal femur (LH), medial height of the distal femur (MH), lateral femoral anteroposterior diameter (LFAP), and medial femoral anteroposterior diameter (MFAP). Two circles were centred on the femoral shaft to determine the long axis of the distal femur. A line passing through the centre of both circles was considered the long axis of the distal femoral shaft. The LFPR and MFPR were determined using a circle-fitting technique in which the femoral condyle was assumed to have a single radius of curvature in flexion from 10° to 160° as described [15, 16, 20]. The line crossing the centre of the femoral posterior circle and perpendicular to the axis of the distal femoral shaft was used to determine the LFAP and MFAP. The distance from the intersection of those lines to the distal femoral condyle was used to determine the LH and MH. The LFPR was divided by the LFAP and multiplied by 100%, and this ratio was defined as the lateral femoral posterior radius ratio (LFPRR). The MFPR was divided by the MFAP and multiplied by 100%, and this ratio was defined as the medial femoral posterior radius ratio (MFPRR) (Fig. 2). The interobserver and intraobserver reliabilities were calculated by using the intraclass correlation coefficient (ICC). To assess intraobserver reliability, each patient was remeasured > 1 week after the initial measurements by the first blinded observer. To determine interobserver reliability, an additional blinded and independent observer repeated the set of measurements.

### Statistical analyses

Statistical analyses were conducted using SPSS software (24, IBM, Chicago, USA). The mean, standard deviation, range and frequency were calculated for continuous variables and percentages. The ICC was calculated to ensure interobserver and intraobserver reliability. According to the normality of the measurements, the Mann-Whitney U test and 2-sample t-test were performed to detect significant differences in all continuous variables, including age, height, weight, BMI, LFPR, MFPR, LH, MH, LFAP, MFAP, LFPRR, and MFPRR, between the ACL-injured group and the control group. The odds ratio (OR) was calculated to determine whether an increased LFPRR and increased MFPRR were risk factors for noncontact ACL injury. A receiver operating characteristic (ROC) curve was used to determine the association between LFPRR and ACL injury and the association between MFPRR and ACL injury. The cut-off was determined at the maximal Youden index with autofit sensitivity and specificity.

Power analysis was performed using G*Power (3.1.9.2, Kiel, Germany) to determine the sample size. According to the preliminary results [11, 12], to achieve a power of 0.95 (a, 0.05; effect size, 0.65), a total of 126 patients (63 per group) were required for this study.

## Results

The measurements of knee osseous morphological characteristics in this study were reliable and reproducible, which is evidenced by the test-retest reliability, with ICC values ranging from 0.870 to 0.989, both within and between subjects.

There was no difference in demographic data, including sex, age, height, weight, and BMI, between the two groups (Table 1). The Mann-Whitney U test and 2-sample t-test revealed significant differences in LFPR, MFPR, MFAP, LFPRR and MFPRR between the ACL-injured group and the control group (*P* < 0.05), but LH, MH, and LFAP did not differ significantly between the two groups. In addition, the LFPR, MFPR, MFAP, LFPRR and MFPRR were greater in the ACL injury group than in the control group (Table 2).

**Table 1:** Subject demographics

	ACL Injury	Control Group	*P* values
Age, y	29.0 ± 8.9	30.4 ± 7.8	0.235
Height, cm	172.7 ± 7.9	171.8 ± 7.0	0.597
Weight, kg	70.7 ± 11.3	69.3 ± 10.5	0.448
BMI, kg/m^2^	23.6 ± 2.4	23.4 ± 2.5	0.593
Sex, male/female	40/34	40/34	

The date of Age, Height, Weight, and BMI were given as the mean and standard deviation. Mann-Whitney U Test was performed to determine if there was a difference between two groups for the Age, Height, Weight, and BMI

**Table 2:** Comparison of the osseous morphologic measurements among groups

variable	ACL Injury	Control Group	*P* values
LFPR, mm	24.0 ± 2.5	22.4 ± 2.5	< 0.001*
LH, mm	21.0 ± 2.7	20.6 ± 2.8	0.408
LFAP, mm	72.6 ± 6.0	71.2 ± 6.3	0.161
MFPR, mm	23.8 ± 2.4	22.5 ± 2.4	0.004*
MH, mm	22.4 ± 2.6	22.0 ± 2.3	0.366
MFAP, mm	70.9 ± 5.8	68.8 ± 6.2	0.039*
LFPRR, %	32.8 ± 1.6	31.5 ± 1.7	< 0.001*
MFPRR, %	33.5 ± 1.7	32.7 ± 1.6	0.007*

All date was given as the mean and standard deviation. Mann-Whitney U Test was performed to detect the significant differences between two groups for the LFPR and MFPR. 2samples t-tests were performed to detect significant differences between the two groups for LH, LFAP, MH, MFAP, LFPRR, and MFPRR. *Significant difference

**Fig. 3:**
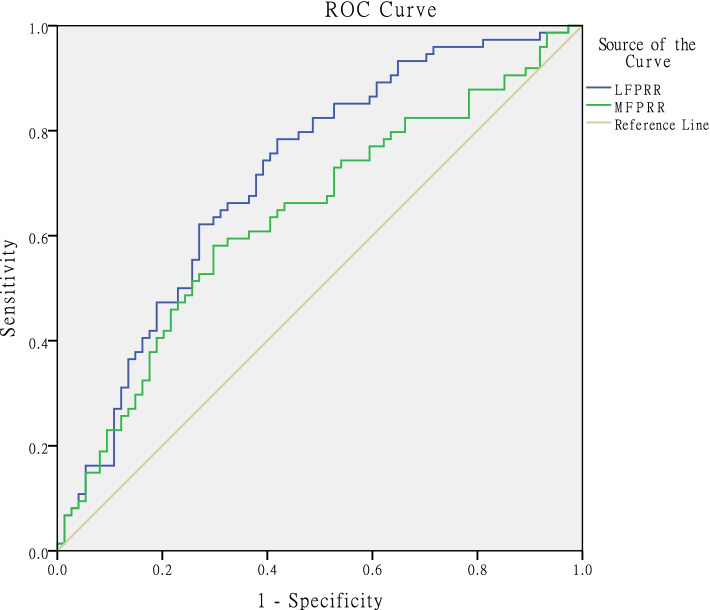
ROC curve analysis was performed to determine the thresholds of LFPRR and MFPRR that were associated with noncontact ACL injury

ROC curve analysis demonstrated that a cut-off of 31.7% (Youden index, 0.365) for the LFPRR yielded a sensitivity of 78.4% and specificity of 58.1% for predicting noncontact ACL injury, and a cut-off of 33.4% (Youden index, 0.284) for the MFPRR yielded a sensitivity of 58.1% and specificity of 70.3% for predicting noncontact ACL injury (Table 3). Additionally, increased LFPRR (> 31.7%) was determined to be a risk factor for noncontact ACL injury (OR = 1.595, 95% CI = 1.281 to 1.985), and increased MFPRR (> 33.4%) was also determined to be a risk factor for noncontact ACL injury (OR = 1.326, 95% CI =1.075 to 1.634). Figure 3 shows the sensitivity and specificity of LFPRR and MFPRR in identifying noncontact ACL injury.

**Table 3:** Cut-off values and their respective AUC of the ROC curve

variable	AUC (95% CI)	Cut-off values, %	Sensibility, %	Specificity, %	*P* values
LFPRR	0.713 (63.0–79.7)	31.7	78.4	58.1	< 0.001*
MFPRR	0.637 (54.7–72.7)	33.4	58.1	70.3	0.004*

The cut-off was determined at the maximal Youden index. *Significant difference

## Discussion

The most important finding of this study is that increased LFPRR and MFPRR were significant risk factors for noncontact ACL injury. In addition, an increased LFPR, increased MFPR, and increased MFAP were significantly associated with noncontact ACL injury. The robust cut-off of 31.7% for the LFPRR could identify patients at risk of ACL injury with a sensitivity of 78.4% and specificity of 58.1%. The robust cut-off of 33.4% for the MFPRR could identify patients at risk of ACL injury with a sensitivity of 58.1% and specificity of 70.3%.

Increased LFPR and MFPR were associated with noncontact ACL injury in our study, and the mechanism of injury may be related to the impact of these femoral osseous morphologies on knee joint kinematics [21–23], which has previously been investigated. Several studies reported that the contour of the posterior femoral condyles had a single radius of curvature [15, 24]. An increased LFPR and increased MFPR indicate that the ACL is likely to suffer greater strain and injury when moving from extension to flexion. The rolling of the lateral femur from its round flexion radius to its flatter part allows a pivot shift of the knee joint [25]. A possible explanation for the increased risk is that an increased LFPR may influence how much rotation the knee allows during a pivot shift movement [26], thereby resulting in a greater pivot shift mechanism. Increased pivoting has been reported to be associated with increased ACL strain and therefore leads to an increased risk of ACL injury [27, 28]. Furthermore, an increased LFPR may result in an increase in the length of the lateral and anterolateral knee structure (the lateral collateral ligament, anterolateral ligament, and anterolateral aspect of the capsule), leading to great anisometry in flexion, the point at which most noncontact ACL injuries occur [11]. However, additional biomechanical and kinematic analyses are needed to investigate how an increased LFPR and increased MFPR elevate the risk of ACL injury by influencing knee joint kinematics.

Pfeiffer et al. determined that an increased lateral femoral condyle ratio is a risk factor for ACL injury [11], where the femoral condyle ratio was defined as the ratio of the femoral posterior condylar depth to the femoral anteroposterior diameter. In our study, the LFPR was greater in the ACL-injured group than that in the control group. It is possible that an increased LFPR contributes to an increased lateral femoral condyle ratio, which is associated with noncontact ACL injury. Although the difference between the lateral femoral condyle and medial femoral condyle has been reported in the existing literature [14, 29], we found that an increased MFPR was also associated with noncontact ACL injury. This observation is important because prior studies focused on the effect of the lateral femoral condyle on ACL injury and ignored the effect of the medial femoral condyle.

The findings of this study showed that an increased MFAP was associated with noncontact ACL injury. A possible explanation for the mechanism is that an increased MFPR leads to an increased MFAP.

The LFPRR and MFPRR were greater in the ACL-injured group than those in the control group. The ROC curve determined that increased LFPRRs and MFPRRs were risk factors for noncontact ACL injury, and LFPRR and MFPRR could be used to identify patients at risk of ACL injury. An increased LFPRR above 31.7% and an increased MFPRR above 33.4% could robustly identify ACL-injured patients. Although the sensitivity and specificity of both diagnostic indicators are moderate, the findings of this study are clinically relevant, as they can aid in the development of screening tools to determine who is at increased risk of noncontact ACL injury and ultimately target this population for intervention programs.

We acknowledge that this study possesses limitations. Each participant underwent a CT scan, which inherently increases risk of radiation exposure. If measurements had been taken using MRI instead of CT, it may have resulted in greater clinical applicability. It is possible that utilization of a healthy control group instead of individuals with tibial plateau fractures may have provided more accurate results. In addition, standard sagittal views of the lateral and medial distal femur were derived from a three-dimensional model of the distal femur, created from DICOM CT images, which were obtained using the image processing software Mimics. Then, three-dimensional rotation was conducted on the models to obtain accurate realignment. This is a complex and expensive method that is more time-consuming than radiographic methods. Despite these limitations, the measurements of the osseous morphological characteristics are associated with knee joint kinematics and are more precise due to three-dimensional model reconstruction and model rotation.

## Conclusions

This study demonstrates that an increased LFPR, increased MFPR and increased MFAP are each associated with noncontact ACL injury. Increased LFPRR and MFPRR are independent risk factors for noncontact ACL injury, and they are clinically relevant for predicting the prevalence of noncontact ACL injury, Clinicians may find this beneficial for identifying susceptible individuals and performing noncontact ACL injury prevention interventions.

## Data Availability

All data and materials are available from the corresponding author upon request.

## Abbreviations

BMI: Body Mass Index
LFPR: Lateral femoral posterior radius
MFPR: Medial femoral posterior radius
LH: Lateral height of distal femur
MH: Medial height of distal femur
LFAP: Lateral femoral anteroposterior diameter
MFAP: Medial femoral anteroposterior diameter
LFPRR: Lateral femoral posterior radius ratio
MFPRR: Medial femoral posterior radius ratio
AUC: Area under the curve
CI: Confidence interval
